# Acid phosphatase 2 (ACP2) is required for membrane fusion during influenza virus entry

**DOI:** 10.1038/srep43893

**Published:** 2017-03-08

**Authors:** Jihye Lee, Jinhee Kim, Kidong Son, Anne-Laure Pham Humg d’Alexandry d’Orengiani, Ji-Young Min

**Affiliations:** 1 Respiratory Viruses Research Laboratory, Discovery Biology Department, Institut Pasteur Korea, Seongnam, Gyeonggi, Republic of Korea

## Abstract

Influenza viruses exploit host factors to successfully replicate in infected cells. Using small interfering RNA (siRNA) technology, we identified six human genes required for influenza A virus (IAV) replication. Here we focused on the role of acid phosphatase 2 (ACP2), as its knockdown showed the greatest inhibition of IAV replication. In IAV-infected cells, depletion of ACP2 resulted in a significant reduction in the expression of viral proteins and mRNA, and led to the attenuation of virus multi-cycle growth. ACP2 knockdown also decreased replication of seasonal influenza A and B viruses and avian IAVs of the H7 subtype. Interestingly, ACP2 depletion had no effect on the replication of Ebola or hepatitis C virus. Because ACP2 is known to be a lysosomal acid phosphatase, we assessed the role of ACP2 in influenza virus entry. While neither binding of the viral particle to the cell surface nor endosomal acidification was affected in ACP2-depleted cells, fusion of the endosomal and viral membranes was impaired. As a result, downstream steps in viral entry were blocked, including nucleocapsid uncoating and nuclear import of viral ribonucleoproteins. Our results established ACP2 as a necessary host factor for regulating the fusion step of influenza virus entry.

Influenza virus is responsible for respiratory diseases that can be severe or even lethal, especially in young children and the elderly^1^. The virus causes annual epidemics and occasional pandemics, and thus represents a threat to human health. Influenza virus is an enveloped virus that belongs to the *Orthomyxoviridae* family and has a genome containing eight negative-sense single strands of RNA^2^. This genome encodes 11 different proteins, two of which—hemagglutinin (HA) and the matrix protein M2—are essential for entry of the viral particle into the host cell^3^,^4^.

Entering the host cell is a crucial step in successful viral infection. Entry of influenza virus can be divided into six sub-steps: attachment, endocytosis, acidification, fusion, uncoating, and nuclear import^5^. The viral membrane-bound glycoprotein HA recognizes sialic acid moieties on the host-cell surface, enabling attachment of the virion. The viral particle is then internalized by endocytosis into an early endosome. This step occurs mostly by a clathrin-mediated process, but macropinocytosis has recently been described as an alternative^6^,^7^. Upon endocytic uptake, the early endosomes become increasingly acidic while maturing into late endosomes^8^. This endosomal acidification drives fusion between viral and endosomal membranes, causing a conformational change of HA to its fusion-active state^9^. At the same time, protons (H^+^) in the acidic endosome are imported into the virion through the M2 ion channel. As a result, the viral ribonucleoprotein complexes (vRNPs) are dissociated from M1 and released into the cytoplasm after fusion. The released vRNPs are imported into the nucleus through a karyopherin-dependent transport mechanism^10^,^11^.

Of the currently available anti-influenza drugs, amantadine and rimantadine target the M2 ion channel whereas oseltamivir and zanamivir target the neuraminidase (NA) protein^12^,^13^,^14^,^15^. Resistance of the virus to one or both the classes of drugs has become a growing concern^16^,^17^. Therefore, host factors essential for viral replication have been considered attractive therapeutic targets to prevent influenza virus infection, because there is no mutational pressure on them to give rise to drug-resistant mutants. These host factors must be identified and their roles in the virus life cycle elucidated to enable the development of novel drugs targeting such host factors. The RNA interference (RNAi) technique allows the identification of host factors involved in viral infections. Over a thousand human genes affecting influenza virus replication have been identified using this technique^18^. However, few follow-up studies have been conducted focusing on the roles of individual identified factors during the viral life cycle.

In this study, we performed cell-based siRNA screens and identified six host factors required for influenza virus replication. Among them, we focused our further studies on the acid phosphatase 2 (ACP2), a lysosomal acid phosphatase. Depletion of ACP2 led to decreased expression of viral proteins and mRNAs. Depletion of ACP2 also decreased the multiple cycle growth kinetics by one log. We also found that knockdown of ACP2 reduced the viral replication of seasonal influenza A and B viruses and avian influenza A viruses (AIVs) of the H7 subtype. Further studies indicated that the mechanism by which ACP2 knockdown reduced viral replication was through inhibition of fusion between endosomal membrane and viral envelope. This reduction in replication was specific to influenza virus and was not observed upon Ebola or hepatitis C virus infection of ACP2-knockdown cells. This is the first report that ACP2 is a crucial cellular protein for the membrane fusion step of the influenza virus entry process.

## Results

### siRNA screen

**Figure 1:**
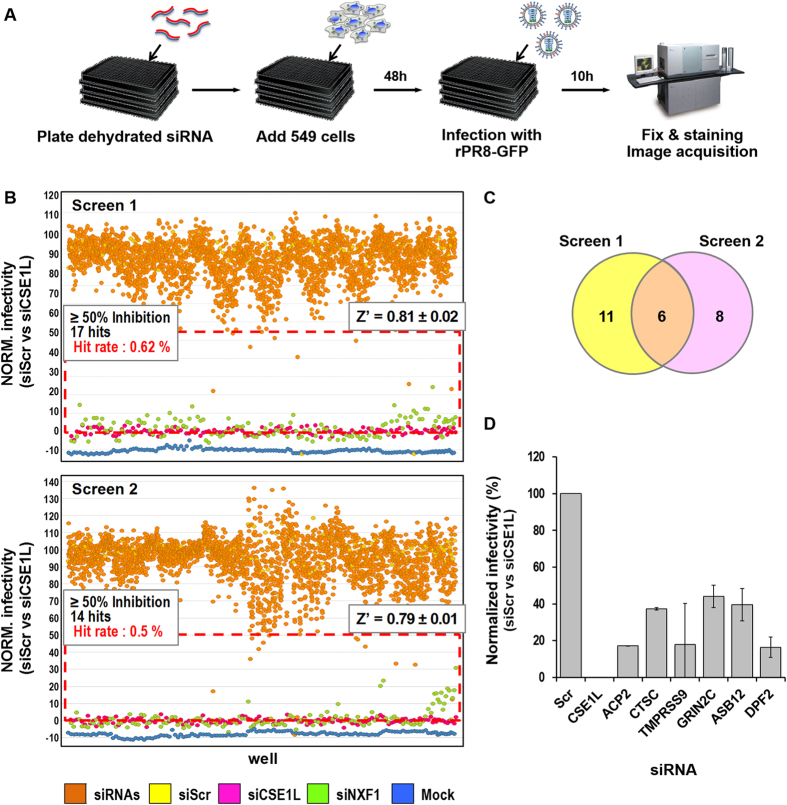
siRNA screening identifies six human genes required for the replication of influenza virus. (**A**) Schematic representation of the screening procedure. (**B**) Scatter plot distribution of the duplicate screening assays. Virus replication was quantified by measuring percentage of GFP-positive cells and normalized to that of siScr-transfected cells and that of siCSE1L transfected cells, which were set 100% and 0%, respectively. The red box indicates hits meeting the selection criteria and the Z’ factors indicated on each diagram are averaged from the duplicated screens of ten 384-well plates. (**C**) Venn diagram of hits validated in duplicate screenings, indicating six overlapping hits representing genes of interest. (**D**) The six siRNAs targeting the indicated genes inhibit the replication of rPR8-GFP virus by more than 50%. Error bars are representative of standard deviation (SD) from duplicate screenings. All reductions are significant (*p* < 0.05, Student’s *t*-test).

To identify host factors required for influenza virus replication, we carried out large-scale siRNA screens using a recombinant IAV (rPR8-GFP) that expresses GFP in infected cells^19^. A siRNA library targeting 2,732 druggable human genes was used to transfect human lung epithelial A549 cells. These cells were subsequently infected with the rPR8-GFP virus and viral replication was monitored by measuring the percentage of GFP-positive cells (Fig. 1A). The screen was optimized using control siRNAs: a non-targeting scrambled siRNA (siScr) was used as a negative control, and siRNAs targeting two human factors, chromosome segregation 1-like (CSElL) and nuclear RNA export factor 1 (NXF1), were used as positive controls. It was previously shown that depletion of CSE1L^20^ or NXF1^21^ reduces infection by inhibiting nuclear import or export, respectively, of influenza vRNPs. Using these screening conditions, more than 80% of siScr-transfected cells were infected (Fig. S1A), while cells transfected with siRNAs against either CSE1L (siCSE1L) or NXF1 (siNXF1) inhibited infection by more than 85% (Fig. S1B).

The screen was conducted in duplicate. The robustness of the assays was demonstrated by Z’ factors greater than 0.5 in both replicates. For hit selection, we normalized the percent infectivity, using the percentage of cells transfected with siScr as 100% and the percentage of cells transfected with siCSE1L as 0%. Within that frame of infectivity values, we selected human genes whose knockdown reduced viral infection by more than 50% (Fig. 1B). Six human genes met this criteria in both of the duplicate screening assays, including acid phosphatase 2 (*ACP2*), cathepsin C (*CTSC*), transmembrane protease serine 9 (*TMPRSS9*), glutamate receptor ionotropic N-methyl D-aspartate 2C (*GRIN2C*), ankyrin repeat and SOCS box-containing 12 (*ASB12*), and double PHD fingers 2 (*DPF2*) (Fig. 1C,D). Of these six gene candidates, *ACP2*^22^, *GRIN2C*^23^, and *DPF2*^24^ were also previously identified in other siRNA screenings, suggesting that our assay was reliable.

### ACP2 is required for IAV replication

**Figure 2:**
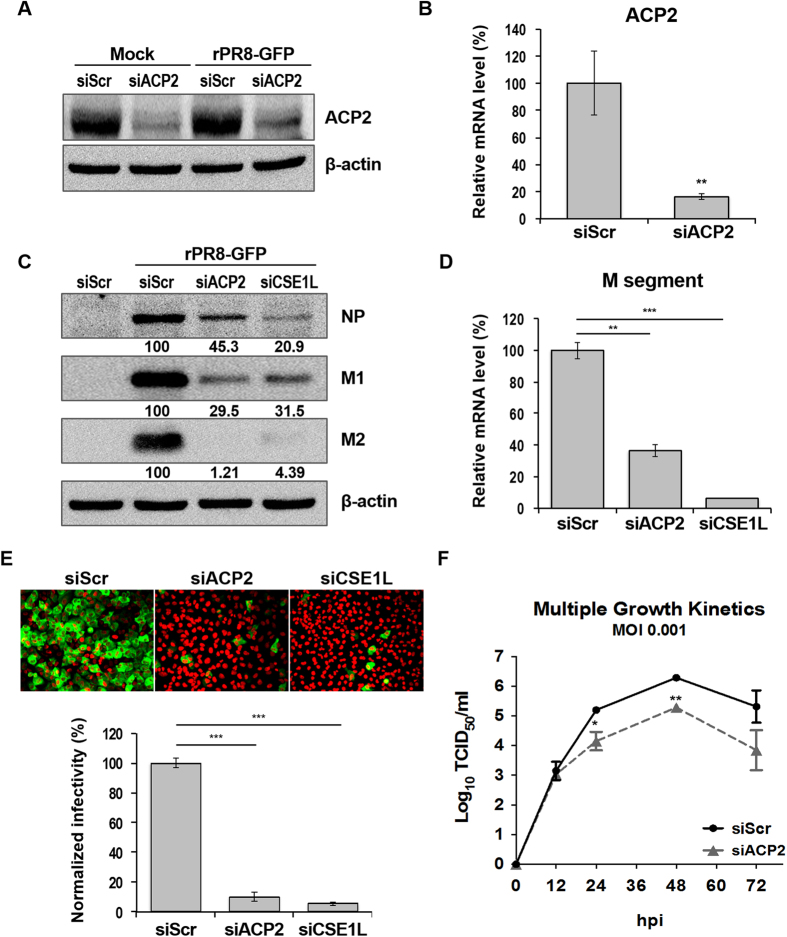
ACP2 depletion inhibits IAV infection. (**A**) Reduction of ACP2 protein expression by siACP2 in both non-infected and infected cells. A549 cells were transfected with indicated siRNAs. After 48 hours, cells were infected with rPR8-GFP virus or not (Mock) for 10 hours and then subject to western blot analyses using the anti-ACP2 and anti-β-actin antibodies. (**B**) Reduced *ACP2* mRNA levels in siACP2-transfected cells. (**C**) Viral protein expression in ACP2-depleted cells. A549 cells were transfected with indicated siRNAs for 48 hours. Cells were then infected with the rPR8-GFP virus at an MOI of 1. After 10 hours, cells were lysed and subject to western blot analyses using the anti-ACP2, anti-NP, anti-M2, anti-M1, and anti-β-actin antibodies. The band density was quantified by Image J software. All values are relative to those of cells transfected with siScr. (**D**) Reduced viral mRNA levels upon ACP2 depletion. A549 cells were transfected with siACP2, siScr, or siSCE1L as indicated prior to infection with rPR8-GFP virus at an MOI of 1. After 10 hours, total RNA was extracted and subject to qRT-PCR. Mean mRNA expression was normalized to that of siScr-transfected cells using the 2ΔCt method. Means from triplicate experiments are given. Error bars represent ± SD. (**E**) ACP2 depletion reduced GFP expression in cells infected with rPR8-GFP virus. A549 cells were transfected for 48 hours with the indicated siRNAs, before infection with the rPR8-GFP virus at an MOI of 1. After 10 hours, cells were fixed and observed with a microplate imaging reader. Quantification of GFP-positive cells was normalized to that of the siScr-transfected cells. Bars show means ± SD from quadruplicated experiments. (**F**) Viral particle production was impeded by ACP2 depletion. A549 cells were transfected with indicated siRNAs for 48 hours before infection with the rPR8-GFP virus at an MOI of 0.001. Supernatant from the infected cells was collected at 12, 24, 48, and 72 hours post-infection. Viral titer was determined by TCID_50_ assays on MDCK cells. Data are represented as the mean ± SD of three independent experiments. Statistical significance between the indicated groups was tested using the Student’s *t*-test; *p < 0.05; **p < 0.01; ***p < 0.001.

We chose *ACP2* for further examination because its knockdown led to the greatest inhibition of rPR8-GFP replication (Fig. 1D). First, we examined the knockdown efficiency of the ACP2-specific siRNA (siACP2) by western blot analysis and quantitative reverse-transcription PCR (qRT-PCR). As shown in Fig. 2A, levels of endogenous ACP2 protein were decreased by siACP2 in both non-infected and infected cells. We also found that siACP2 significantly reduced the *ACP2* mRNA level (Fig. 2B). Next, we confirmed the effect of ACP2 depletion on the replication of rPR8-GFP virus. Knockdown of ACP2 reduced expression of the viral nucleoprotein (NP), M1, and M2 proteins, as well as the M1 mRNA level (Fig. 2C and D). Confocal microscopy also demonstrated that GFP expression in cells after addition of rPR8-GFP was decreased up to 90% by ACP2 depletion compared to GFP expression in siScr-transfected cells (Fig. 2E). Finally, we investigated whether the depletion of ACP2 affected the multiple cycle growth of rPR8-GFP virus. To this end, siACP2- or siScr-transfected A549 cells were infected at a low multiplicity of infection (MOI) of 0.001, and supernatant was collected at different time points after infection and used for a TCID_50_ assay. ACP2 depletion reduced the viral particle production by one log compared to that of siScr-transfected cells (Fig. 2F). Together, these results demonstrate that ACP2 was necessary for IAV replication.

### ACP2 is necessary for the replication of influenza A and B viruses

**Figure 3:**
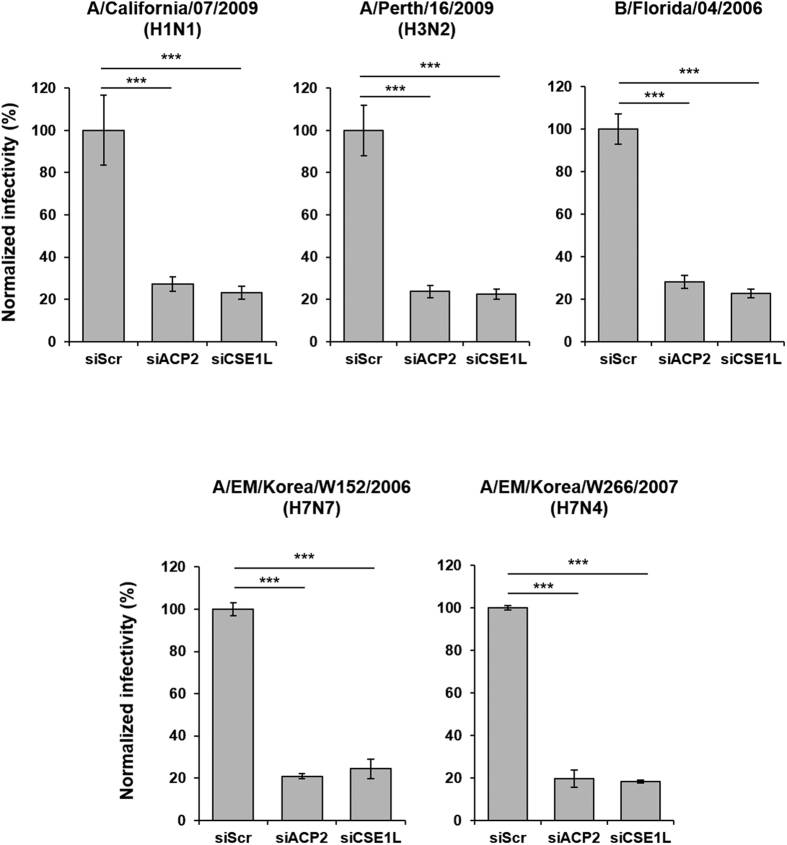
ACP2 is necessary for the replication of various wild-type influenza A and B viruses. A549 cells were transfected with siScr, siACP2, or siCSE1L. After 48 hours, cells were infected with the indicated viral strains at an MOI of 1 for 10 hours. The cells were stained with an anti-NP antibody and analyzed by confocal microscopy. Virus replication was determined by calculating the percentage of NP-expressing cells and normalized to that of the siScr-transfected cells. Bars show means ± SD from quadruplicated experiments. Statistical significance between the indicated groups was tested using the Student’s t-test with a threshold of ***p < 0.001.

We used the rPR8-GFP virus for the siRNA screening as well as to validate the effect of ACP2 depletion on viral replication. However, rPR8-GFP is a lab-adapted strain and recombinant virus; therefore, we also assessed whether ACP2 is required for replication of a range of contemporary circulating influenza virus strains: A/California/07/2009 (H1N1), A/Perth/16/2009 (H3N2), and B/Florida/04/2006. In addition, we tested the replication of two AIVs of the H7 subtype, A/EM/Korea/W266/2007 (H7N4) and A/EM/Korea/W152/2006 (H7N7), because there have been occasional case reports of direct animal-to-human transmission causing human H7 influenza virus infections. As shown in Fig. 3, ACP2 knockdown greatly reduced the infectivity of all tested strains compared with the siScr control. These results confirm that ACP2 is required for the efficient replication of various influenza A and B virus strains.

### ACP2 is not required for the attachment or acidification steps of influenza virus entry

**Figure 4:**
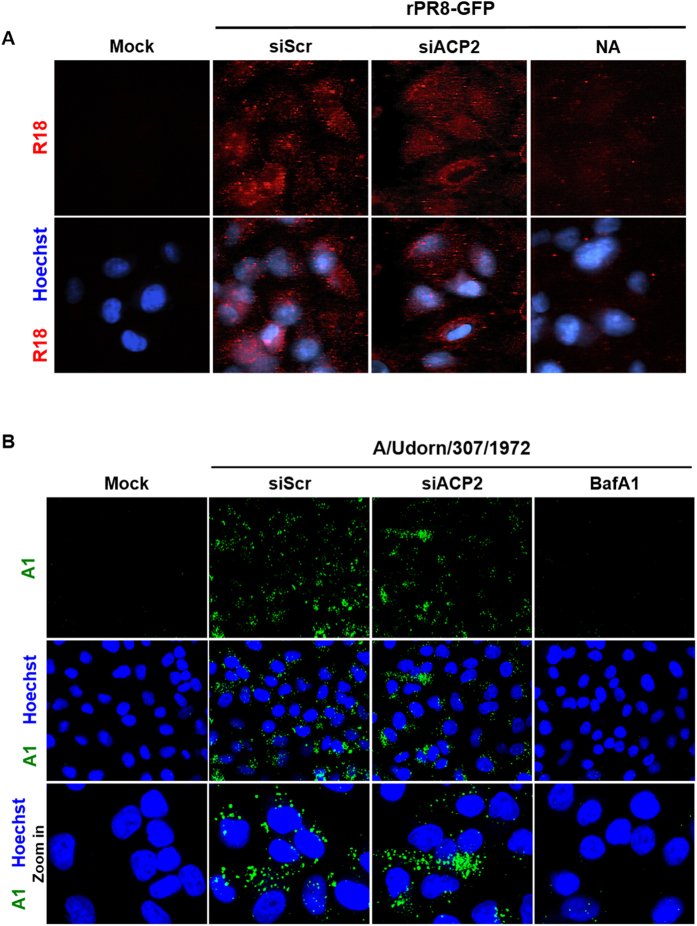
ACP2 knockdown does not affect attachment of the virus or acidification of endosomes. (**A**) ACP2 does not affect attachment of the viral particles to cells. A549 cells transfected with the indicated siRNAs were incubated with or without 0.25 U/ml neuraminidase for 4 hours then inoculated with an R18-labeled virus at an MOI of 100 for 1 hour at 4 °C. Cells were then fixed for observation. (**B**) A549 cells were transfected with the indicated siRNAs at 37 °C for 48 hours prior to infection with A/Udorn/307/1972 (H3N2) at an MOI of 100 at 4 °C for 1 hour. After inoculation, cells were incubated at 37 °C for an additional hour in the presence or absence of 0.1 μM bafilomycin A1 (BafA1). Cells were then fixed and stained with an anti-HA antibody that specifically recognizes the acid-induced conformation of the HA protein of H3.

ACP2 is a lysosomal protein localized to both the lysosome and the extracellular environment^25^,^26^. Because most of the steps in influenza virus entry occur in endolysosomes^27^, we hypothesized that ACP2 might play a crucial role in influenza virus entry. To test this hypothesis, we divided the entry process into five sub-steps—attachment, endosomal acidification, membrane fusion, uncoating, and nuclear import of vRNPs—and monitored each step sequentially by visualizing virus particles or viral proteins. To examine the effect of ACP2 depletion on attachment of the virus to host cells, siRNA-transfected cells were infected with octadecyl rhodamine B chloride (R18)-labeled rPR8-GFP virus, which can be visualized by confocal microscopy. As a positive control, siScr-transfected cells were treated with neuraminidase before viral infection. Neuraminidase cleaves the glycosidic linkages of sialic acid molecules, and thus prevents attachment of the viral particle to the cell surface^28^. After 1 hour of inoculation with the labeled virus, unbound viral particles were removed and cell-bound viral particles were visualized. An identical fluorescence pattern was observed between ACP2-depleted cells and control cells transfected with siScr, whereas reduced R18 signal was detected in cells treated with neuraminidase (Fig. 4A). These data show that ACP2 was not crucial for the attachment step of influenza virus entry.

We then assessed the acidification step using a monoclonal antibody A1, which is specific for the acid-induced fusion-active conformation of HA protein^29^. Bafilomycin A1 (BafA1) was used as a positive control, because it is an inhibitor of the vacuolar ATPase required for endosome acidification and thus blocks the conformational change of HA^30^. The fusion-active conformation of HA was detected in both siScr- and siACP2-transfected cells, but barely observed in BafA1-treated cells (Fig. 4B), suggesting that the acidification step was not affected by ACP2 depletion. As such, we concluded that ACP2 is not required for the first two steps of influenza virus entry, *i.e.*, attachment and acidification.

### ACP2 plays a crucial role in the membrane fusion step of influenza virus entry

**Figure 5:**
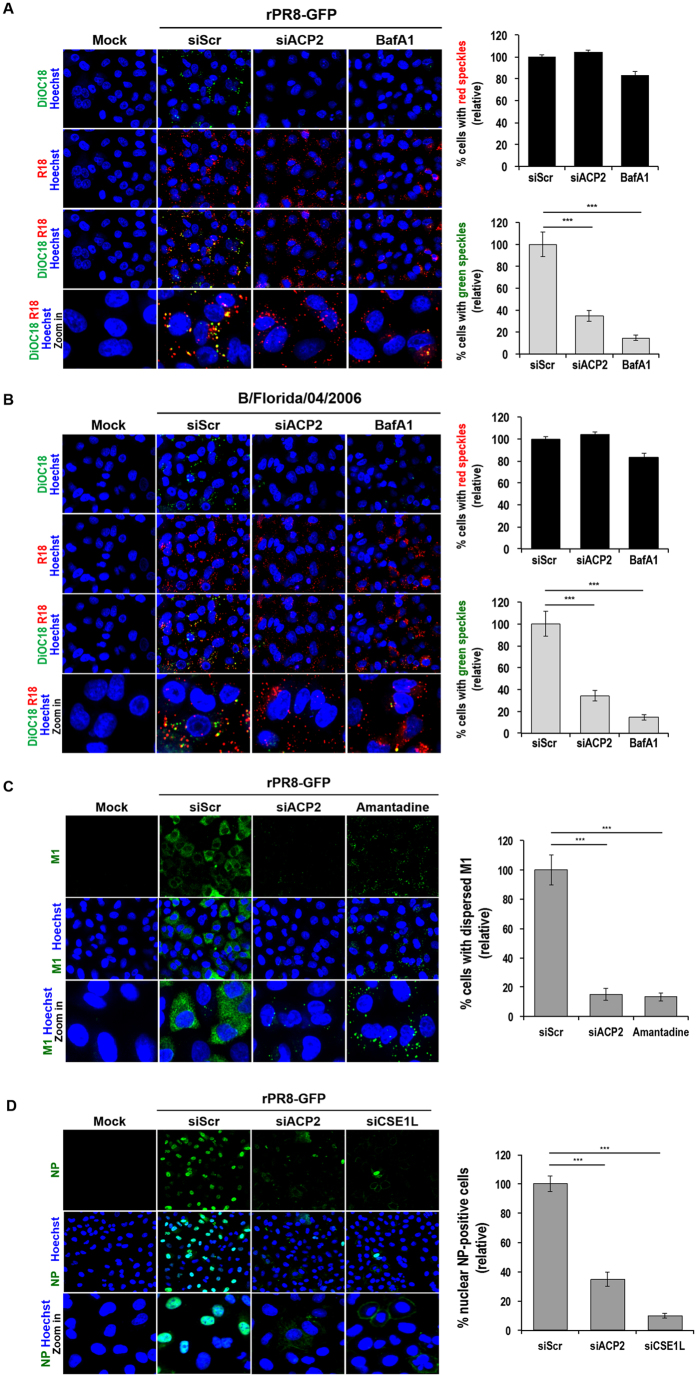
ACP2 is crucial for membrane fusion during entry of influenza A and B viruses. (**A** and **B**) ACP2 knockdown impaired the fusion step between viral and endosomal membranes. A549 cells were transfected with the indicated siRNAs at 37 °C for 48 hours prior to infection with viruses rPR8-GFP (**A**) or B/Florida/04/2006 (**B**) that had been labeled with R18 and DiOC18 at an MOI of 100 at 4 °C for 1 hour. Cells were then washed with PBS and incubated at 37 °C for an additional 1.5 hours with or without 0.1 μM of bafilomycin A1 (BafA1) prior to fixation. The number of the cells with red (R18) or green (DiOC18) speckles was quantified using in-house-developed image-mining (IM) software and normalized to siScr control cells (right panel). (**C**) Cytoplasmic M1 expression was decreased in ACP2-depleted cells. A549 cells were transfected with the indicated siRNAs at 37 °C for 48 hours prior to rPR8-GFP virus infection at an MOI of 100 at 4 °C. Cells were then further treated with 0.5% DMSO or 1 mM amantadine for 3 hours at 37 °C. Cells were then fixed and stained with the anti-M1 antibody. The number of the cells with dispersed M1 in cytoplasm was quantified and normalized as described in the legend to panel A and B (right panel). (**D**) vRNP import into the nucleus is impeded by knockdown of ACP2. A549 cells were transfected with the indicated siRNAs at 37 °C for 48 hours prior to rPR8-GFP infection at an MOI of 100 at 4 °C. Cells were then incubated at 37 °C for 3.5 hours before staining with the anti-NP antibody. The number of the nuclear NP-positive cells was quantified and normalized as described in the legend to panel A and B. Bar graphs show means ± SD from three independent experiments. Statistical significance between the indicated groups was tested using the Student’s t-test with a threshold of: ***p < 0.001.

We next investigated the effects of ACP2 knockdown on the membrane fusion step. To observe the fusion step, we used an assay based on the fluorescence dequenching of the lipophilic dyes 3,3′-dioctadecyl-5,5′-di(4-sulfophenyl)-oxacarbocyanine, sodium salt (SP-DiOC18) and R18 as previously described by Banerjee *et al*.^29^. A virion labeled with both SP-DiOC18 and R18 dyes emits only red fluorescence from the R18 dye, because the green fluorescence from the SP-DiOC18 dye is quenched by R18 in close proximity within the viral envelope. However, when the viral envelope merges with the endosomal membrane and allows both dyes to diffuse through the endosomal membrane, the green SP-DiOC18 signal increases due to dequenching. BafA1 prevents acidification of endosomes, thus blocking the membranes from fusing. Upon infection by labeled rPR8-GFP virus, both SP-DiOC18 and R18 were detected as speckles of green and red fluorescence, respectively, in siScr-transfected cells, indicating membrane fusion (Fig. 5A), whereas few green speckles were detected in BafA1-treated cells. In siACP2-transfected cells, there were fewer green speckles, similar to what was seen in BafA1-treated cells, indicating that fusion between the endosomal membrane and viral envelope did not occur. The membrane fusion was significantly reduced up to 65% and 85% by ACP2 knockdown and BafA1 treatment, compared with siScr control, respectively (Fig. 5A, right panel). We also determined that the depletion of ACP2 inhibited the fusion process during entry of an influenza B virus. Depletion of ACP2 significantly reduced fusion following infection with B/Florida/4/2006 (Fig. 5B). These results demonstrate that ACP2 is required for the fusion of membranes during entry of influenza A and B viruses.

We next observed the effects of ACP2 knockdown on the downstream steps, namely uncoating of nucleocapsid and nuclear import of the vRNPs. To detect the uncoating step, we monitored the cytosolic levels of the M1 protein, as M2-mediated viral acidification leads to a dissociation of vRNPs from M1, resulting in the release of M1 into the cytoplasm. As a control, we treated cells with the M2 inhibitor amantadine, thereby inhibiting the low-pH-induced dissociation of M1 and vRNPs^12^,^31^. While M1 proteins in siScr-transfected cells were distributed throughout the cytoplasm, M1 proteins in siACP2-depleted cells displayed a scattered, punctate pattern, similar to that observed in cells treated with amantadine (Fig. 5C). The degree of the uncoating was quantified by measuring the cells with dispersed M1 signal. ACP2 depletion inhibited the uncoating by than 80%, compared with siScr control (Fig. 5C, right panel).

Finally, we investigated whether nuclear import of vRNPs into the nucleus was affected when ACP2 was depleted by observing the subcellular localization of NP. Knockdown of CSE1L was used as a control, as previous studies showed that depletion of CSE1L prevented the nuclear import of vRNPs^20^. In siScr-transfected cells, viral NP was located primarily in the nuclei, indicating that vRNPs were efficiently imported into the nucleus (Fig. 5D). However, nuclear NP was barely detectable following ACP2 or CSE1L knockdown. More than 60% of vRNPs import in siACP2-depleted cells was decreased compared to siScr-transfected cells (Fig. 5D, right panel). These data indicated that the inhibition of membrane fusion induced by ACP2 depletion subsequently impaired downstream viral entry steps. Taken together, our results suggest that influenza A and B viruses require ACP2 for viral entry, specifically at the membrane fusion step.

### ACP2 is not required for the replication of other viruses such as Ebola and Hepatitis C

Our data so far suggested that ACP2 is a cellular factor required for the fusion of the influenza viral envelope with the endosomal membrane. After endocytosis, influenza viruses are trafficked into the acidic environment of endosomal compartment, where they undergo pH-dependent membrane fusion. Since many enveloped viruses share the common entry steps and pathways^32^, like influenza viruses, it was of interest to investigate whether ACP2 was required for those viruses. Ebola virus and hepatitis C viruses are both enveloped, which undergo the same pH-dependent membrane fusion^33^,^34^, therefore we chose these two viruses for the comparison.

**Figure 6:**
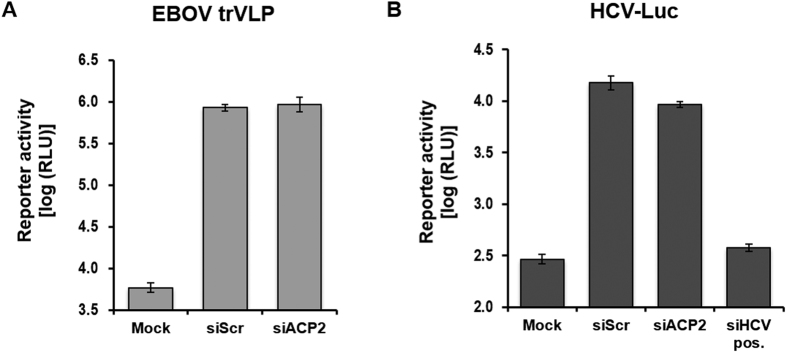
ACP2 is not required for the replication of Ebola or hepatitis C viruses. ACP2 depletion has no effect on the replication of Ebola virus (**A**) or hepatitis C virus (**B**). 293 T cells or Huh7.5 cells were transfected with the corresponding siRNAs and then infected with Ebola-trVLPs or HCV-*luc*, respectively, after 48 hours. At 72 hours post-infection, replication of each virus was quantified by measuring *Renilla* luciferase activity. Data are represented as the mean ± SD of three independent experiments.

To investigate whether ACP2 was required for Ebola virus replication, we used an Ebola transcription- and replication-competent virus-like particle (trVLP) system. trVLPs containing a minigenome encoding a reporter gene are able to infect target cells, delivering the minigenome into the cells. Using the trVLP system previously described by Watt and colleagues^35^, in which the *Renilla* luciferase is used as the reporter gene, replication and transcription of the minigenome in target cells can be detected by measuring luciferase reporter activity. As depicted in Fig. 6A, no difference in reporter activity was observed between ACP2-depleted and siScr-transfected cells. We also examined the effect of ACP2 knockdown during HCV infection using an HCV reporter virus expressing the *Renilla* luciferase (HCV-luc)^36^. Our results show that depletion of ACP2 had no significant effect on HCV replication, whereas a positive control siRNA targeting the 5′-untranslated region of the HCV genome completely abolished replication compared to siScr-transfected cells (Fig. 6B). In summary, these results suggest that ACP2 is a crucial entry factor required specifically by the influenza virus.

## Discussion

RNAi is a powerful tool to understand the relationship between a host and a pathogen. Utilizing this technique, seven research groups identified over a thousand host factors regulating influenza virus replication^18^. However, discrepancies exist regarding the identified host factors, and only 20 genes overlapped as among the results from three independent screens. There were no host factors common to all seven screens. Parameters such as the cell lines, viral strains, RNAi library, experimental conditions, readout, or selection criteria likely affect the outcomes of different screening assays^18^,^37^,^38^. In the present study, we used an IAV that contains a GFP-encoding gene fused to the non-structural (NS) gene segment to facilitate readout for screening. A549 cells were used because they are human lung epithelial cells and thus a relevant cell line for the study of influenza virus, which causes respiratory disease. We chose an siRNA library that specifically targets druggable genes because these are expected to be potential therapeutic candidates, and performed the screening twice for validation of the hits. From our screen, six human genes were identified as host factors required for the replication of influenza virus. Among those genes, *ACP2*^22^, *GRIN2C*^23^, and *DPF2*^24^ were already reported to be important factors for influenza replication, and their presence in our results confirmed the robustness of our method. We also identified two novel host factors, *CTSC* and *TMPRSS9*, which have not previously been reported to be associated with influenza replication and represent opportunities for future study.

In this study, we focused our analysis on *ACP2* because silencing of this gene showed the greatest inhibition of viral replication. Although this protein was previously identified as a human factor required for influenza virus infection^22^, the precise role of ACP2 in the viral life cycle was not elucidated. Our present study provides additional evidence for the requirement of ACP2 in influenza virus infection. ACP2 knockdown decreased expression of viral proteins and mRNA as well as the multiple cycle growth of the lab-adapted rPR8-GFP virus. Importantly, we also demonstrated the applicability of this finding to various wild-type influenza viruses, including currently circulating seasonal influenza A and B viruses and AIVs of the H7 subtype. These results suggest that ACP2 plays a crucial role in the replication of influenza viruses.

ACP2 is a lysosomal acid phosphatase that was initially discovered in the lysosomal compartment and is widely used as a lysosome marker^25^,^26^,^39^. While the role of ACP2 in mammalian cells has been described, its specific role in influenza virus-infected cells remained unknown. The localization of ACP2 led us to hypothesize that it may play a role during viral entry. Entry of influenza virus can be divided into multiple steps and several of these, from endocytosis to membrane fusion, take place in the endolysosomal system. Therefore, we dissected and analyzed the steps of influenza virus entry in ACP2-depleted cells. Based on our results, we have concluded that ACP2 depletion did not impact the attachment of the viral particle and endosomal acidification. The new insight gained in our study is that ACP2 regulates the viral entry process at the level of membrane fusion. This finding is further supported by the disruption of downstream steps including uncoating of the viral nucleocapsid and import of vRNPs into the nucleus.

Overall, our work has revealed the role of ACP2 in influenza virus infection. Additional investigations into the interacting partners of ACP2 could be useful for understanding the network of host-pathogen interactions that drive the early steps of influenza virus infection and replication. We examined the interaction of endogenous ACP2 with viral HA or M2 in infected cells, because these two viral transmembrane proteins play roles in the entry of the viral particle into target cells^10^. The results from our immunoprecipitation assay demonstrated that ACP2 did not directly interact with HA or M2 (data not shown). Fusion of the viral envelope with the endosomal membrane is mediated by the HA2 subunit that is produced by proteolytic cleavage of the HA protein^40^. The drop in pH during endosome maturation is required for the conformational change of HA2, which exposes the fusion peptide and leads to membrane fusion^41^. The acidic environment in the lysosome is also important for the hydrolase activity of ACP2. ACP2 is converted from a membrane-bound precursor to a soluble, mature phosphatase by proteolytic cleavage after delivery to lysosomes^42^. In addition, ACP2 hydrolyzes various phosphomonoesters at an acidic pH of 3.5–5.0 *in vitro*^43^. Interestingly, ACP2 was not identified in a previous siRNA screen that used a modified influenza viral strain in which the region encoding the HA protein was replaced with that encoding the *Renilla* luciferase^20^. Based on the previous studies and our data, we could hypothesize that the enzymatic activity of ACP2 may affect, directly or indirectly, the fusogenic state of HA following endosomal acidification.

In Golgi, newly synthesized lysosomal proteins are modified with mannose-6-phosphate (Man6P) residues that serve as recognition markers for the transport of the lysosomal proteins to the endosomal/lysosomal compartment. After arrival of the lysosomal proteins in lysosome, the Man6P recognition maker is dephosphorylated^44^,^45^,^46^. The study of Makrypidi *et al*. demonstrated that ACP2 dephosphorylates the Man6 containing-lysosomal proteins in concert with ACP5^39^. They identified that a lysosomal Niemann-Pick C2 (NPC2) is one of the substrates of ACP2 and ACP5. The NPC proteins, NPC1 and NPC2, play an important role in maintenance of intracellular cholesterol homeostasis by regulating the transport of cholesterol from late endosomes/lysosomes to other cellular compartments^47^,^48^. Indeed, highly elevated amount of cholesterol in lysosomes was observed in *Acp2/Acp5*^*−/−*^ hepatocytes^39^. Interestingly, an interferon-inducible transmembrane protein 3 (IFITM3) induced cholesterol accumulation in late endosome, which blocks the fusion between intraluminal virion-containing vesicles and endosomal membranes and thereby inhibits the entry of vesicular stomatitis virus^49^. Our data, together with those from other studies, are able to propose one model that impaired removal of Man6P residues from NPC2 by ACP2 depletion may disrupt intracellular cholesterol homeostasis and thereby block influenza viral entry, although further experiments are required to elucidate the model.

Our work has demonstrated that ACP2 was indeed crucial for influenza virus replication and has shown the specificity of ACP2 for influenza viruses, in contrast to other RNA viruses such as Ebola virus and HCV. By identifying the role of ACP2 in membrane fusion during influenza virus entry, our study has provided new insight into the complex network of virus-host interactions during viral entry. This is the first report of the necessity of the host cellular factor ACP2 in influenza virus entry, and suggests that ACP2 may be an attractive target for the development of pan-influenza therapeutics.

## Materials and Methods

### Cells and viruses

Madin-Darby canine kidney cells (MDCK, CCL-34), A549 adenocarcinomic human lung epithelial cells (CCL-185), and HEK 293 T/17 cells (CRL-11268) were purchased from the American Type Culture Collection (ATCC). Cell lines were maintained in Dulbecco’s Modified Eagle Medium - High Glucose (DMEM-HG, WelGene) supplemented with 10% fetal bovine serum and 1% antibiotic-antimycotic solution (Life Technologies). Huh7.5 cells were a kind gift of Dr. Marc Windisch (Hepatitis Research Laboratory, Institut Pasteur Korea) and were maintained in DMEM-HG supplemented with 10% FBS, 2 mM L-glutamine, 1% MEM Non-Essential Amino Acids, and 1% antibiotic-antimycotic solution (Life Technologies). The recombinant influenza A/Puerto Rico/8/1934 (H1N1) virus carrying a green fluorescence protein (GPF) in the NS gene segment (rPR8-GFP) was kindly provided by Prof. Adolfo García-Sastre (Mount Sinai School of Medicine, New York, USA)^19^. Influenza virus strain A/Udorn/307/1972 (H3N2) was rescued by reverse genetics, while A/California/07/2009 (H1N1), A/Perth/16/2009 (H3N2), and B/Florida/04/2006 were obtained from the Korean Center for Disease Control and Prevention. Influenza A strains A/EM/Korea/W266/2007 (H7N4) and A/EM/Korea/W152/2006 (H7N7) were a kind gift of Prof. Young Ki Choi (Chungbuk National University, Cheongju, South Korea). All viruses were grown in embryonated chicken eggs. The Ebola trVLP system was a kind gift of Dr. Thomas Hoenen (National Institute of Allergy and Infectious Diseases, MT, USA). Hepatitis C virus reporter virus, which was the genotype 2a virus J6/JFH1 with the *Renilla* luciferase gene inserted into the nonstructural protein 5A gene (HCV-*luc*) was a kind gift of Prof. Jens Bukh (University of Copenhagen and Hvidovre hospital, Hvidovre, Denmark). Viral stock titers for influenza strains were determined by hemagglutination and plaque assays. TCID_50_ was performed using MDCK cells to determine virus titers during the multiple growth kinetics assay as previously described^50^.

### siRNA screening

Screening was performed using the Human Druggable ON-TARGETplus siRNA library, targeting 2,732 annotated genes (#G-104605, Dharmacon). A549 cells were seeded in 384-well plates (4 × 10^3^ cells/well) in medium without antibiotics and transfected with 10 nM of pooled siRNAs, using 0.2% DharmaFECT1 transfection reagent (Dharmacon). Non-targeting siRNA siScr, siNXF1 and siCSE1L, were used as controls (Dharmacon). At 48 hours post-transfection, media was removed and cells were infected with the rPR8-GFP strain at an MOI of 5 for 1 hour at room temperature (RT). Viral inoculum was removed and Opti-PRO serum-free medium was added (Gibco). At 9 hours post-infection, cells were fixed with 4% paraformaldehyde (PFA, Sigma-Aldrich) containing 2.5 μg/mL Hoechst 33342 for 30 minutes at RT. Cells were imaged using a high-throughput confocal fluorescence imaging system (Evotec Technologies High-Throughput Cell Analyzer Opera, Perkin Elmer) and analyzed with a previously described in-house image mining (IM) platform^51^. The screen was conducted in duplicate. The scatter plot was generated using TIBCO Spotfire 4.5.0 (TIBCO Software Inc.). The Z factor was defined by the means and standard deviations of the percentage of cells infected in the positive and negative controls, and is used to assess the quality of a screening assay^52^.

### Immunofluorescence assay

Following infection and/or transfection, cells were fixed with 4% PFA in phosphate buffered saline (PBS) for 30 minutes at RT, then permeabilized with 0.25% Triton X-100 for 30 minutes (Sigma-Aldrich). Cells were then incubated with 1 μg/ml anti-influenza A virus NP antibody (AA5H, Abcam) or anti-influenza B virus NP antibody (B017, Abcam) for 1 hour at 37 °C. After three washes with PBS, cells were incubated with Alexa 488-conjugated goat anti-mouse IgG (H + L) secondary antibody and Hoechst 33342 (Life Technologies) at a working dilution of 1/4000 for 1 hour at RT. Cells were then washed three times with PBS before analyses.

### Western blot analysis

Following infection and/or transfection, cells were lysed with cell lysis buffer (Cell Signaling Technology) supplemented with protease inhibitor cocktail (Cell Signaling Technology) and 1 mM phenylmethylsulfonyl fluoride (Sigma-Aldrich). Proteins were separated by sodium dodecyl sulfate-polyacrylamide gel electrophoresis and transferred onto nitrocellulose membranes (Bio-Rad). Membranes were then washed with Tris-buffered saline containing 0.2% Tween-20 (TBST). Following blocking with a 5% milk-TBST solution for 1 hour, membranes were incubated with one of the following primary antibodies at 4 °C overnight: mouse monoclonal anti-influenza A virus NP antibody (AA5H, Abcam), anti-influenza A virus M2 mouse monoclonal antibody, rabbit polyclonal anti-acid phosphatase 2 antibody (Abcam), anti-actin mouse monoclonal antibody (Sigma-Aldrich), or anti-influenza A virus M1 antibody (generated from M2-1C6-4R3 hybridoma cells [HB64] purchased from the ATCC). Membranes were then washed three times with TBST before incubation with HRP-conjugated anti-mouse or HRP-conjugated anti-rabbit antibodies (Cell Signaling) for 1 hour at RT. After three washes with TBST, blots were revealed by enhanced chemiluminescence (ECL) detection reagent (GE Healthcare).

### qRT-PCR

Total RNA was extracted from siRNA-transfected cells using Trizol according to the manufacturer’s instructions (Invitrogen). To generate cDNA, 0.5 μg of total RNA was used with the TOPscript^TM^ RT DryMIX containing oligo-dT primers (Enzynomics, Korea). Maxima SYBR Green qPCR Master Mix (Thermo Fischer Scientific) was used with the following primers sets: M-F: 5′-TCAGGCCCCCTCAAAGCCGA-3′, M-R: 5′-GGGCACGGTGAGCGTGAACA-3′, α-tubulin-F: 5′-GCCTGGACCACAAGTTTGAC-3′, and α-tubulin-R: 5′-TGAAATTCTGGGAGCATGAC-3′, following the manufacturer’s instructions. Primers for ACP2 were purchased from Bioneer. Levels of ACP2 and viral M mRNAs were normalized to the expression of α-tubulin using the 2ΔCt method^53^. Data are represented as relative expression to that of the siScr-transfected cells.

### trVLP assay

The trVLP assay was conducted as previously described^35^. To generate trVLPs, 293 T cells, referred to as producer cells or p0 cells, were transfected with four different expression plasmids encoding each component of the vRNP of Ebola virus, as well as a minigenome plasmid encoding the *Renilla* luciferase reporter and the remaining viral proteins. At 72 hours post-transfection, the trVLP-containing supernatant of p0 cells was transferred onto 293 T cells, referred to as target cells. The target cells had been transfected 48 hours previously with siScr or siACP2, then transfected 24 hours earlier with expression plasmids for the vRNP of Ebola virus. The minigenome was delivered to the target cell by infection with the trVLPs. At 72 hours after infection, reporter activity in the target cells was measured with a multi-label plate reader (Victor X3; Perkin Elmer).

### Entry assays

Entry assays were carried out as previously described^29^, with some modifications. For synchronized infection, cells were adsorbed with virus at 4 °C for 1 hour. All images were acquired with a confocal fluorescence imaging system (LSM5, Zeiss or Opera, Perkin Elmer). The acquired images were quantified by in-house-developed IM platform.

#### Binding assay

A549 cells were transfected with siRNAs as described earlier and infected with the rPR8-GFP virus at an MOI of 100 at 4 °C for 1 hour. The virus had previously been labeled with 0.4 μM of the fluorescent dye R18 (Life technologies). After washing with cold PBS to remove the residual virus particles, cells were then fixed with 4% PFA containing 2.5 μg/ml Hoechst 33342 and observed under a confocal microscope.

#### Acidification assay

A549 cells were transfected with siRNAs and infected with A/Udorn/307/1972 (H3N2) virus at an MOI of 100 at 4 °C for 1 hour. After washing cells with cold PBS, serum-free media was added and cells were incubated at 37 °C for 1 hour. Cells were then fixed with 4% PFA and permeabilized with 0.25% Triton X-100. Cells were stained with an anti-HA monoclonal antibody, generated from A1 hybridoma cells (a kind gift of Dr. Yohei Yamauchi, ETH Zurich, Zurich, Switzerland). Cells were then incubated for 1 hour with Alexa 488-conjugated goat anti-mouse IgG secondary antibody and Hoechst 33342 prior to microscopy.

#### Fusion assay

A549 cells were transfected with siRNAs and infected with rPR8-GFP virus at an MOI of 100 at 4 °C for 1 hour. The virus had been previously labeled with fluorescent dyes R18 and SP-DiOC18 (Life technologies). After washing cells with cold PBS, serum-free media was added and cells were incubated for 1.5 hours at 37 °C. Cells were then fixed with 4% PFA containing 2.5 μg/ml of Hoechst 33342 before microscopic observation.

#### Uncoating assay

A549 cells were transfected with siRNAs and infected with rPR8-GFP virus at an MOI of 100 at 4 °C for 1 hour. After washing cells with cold PBS, serum-free media was added and cells were incubated for 3 hours at 37 °C. Cells were then fixed with 4% PFA and permeabilized with 0.25% Triton X-100. Cells were further incubated with the previously mentioned anti-M1 monoclonal antibody at 37 °C. After 1 hour, cells were incubated with Alexa 488-conjugated goat anti-mouse IgG secondary antibody and Hoechst 33342 prior to microscopic observation.

#### Nuclear import assay

A549 cells were transfected with siRNAs and infected with rPR8-GFP virus at an MOI of 100 at 4 °C for 1 hour. After washing cells with cold PBS, serum-free media was added and cells were incubated for 3.5 hours at 37 °C. Cells were then fixed with 4% PFA, permeabilized with 0.25% Triton X-100, and then stained with the anti-NP monoclonal antibody. Cells were further incubated with Alexa 488-conjugated goat anti-mouse IgG secondary antibody and Hoechst 33342 for 1 hour.

## Additional Information

**How to cite this article:** Lee, J. *et al*. Acid phosphatase 2 (ACP2) is required for membrane fusion during influenza virus entry. *Sci. Rep.* **7**, 43893; doi: 10.1038/srep43893 (2017).

**Publisher's note:** Springer Nature remains neutral with regard to jurisdictional claims in published maps and institutional affiliations.

## Supplementary Material

Supplementary Figure 1
