# Supplementary material for: Acid phosphatase 2 (ACP2) is required for membrane fusion during influenza virus entry
Source: Sci Rep. 2017 Mar 8;7:43893. doi: 10.1038/srep43893 (PMC5341025; doi:10.1038/srep43893)
Supplement: Supplementary Figure 1 [file srep43893-s1.pdf]

Acid phosphatase 2 (ACP2) is required for membrane fusion during influenza virus entry

Jihye Lee[1], Jinhee Kim[1], Kidong Son[1], Anne-Laure Pham Hung d'Alexandry d'Orengiani[1] and Ji-Young Min[1*]

[1]Respiratory Viruses Research Laboratory, Discovery Biology Department, Institut Pasteur Korea, Seongnam, Gyeonggi, Republic of Korea

*Corresponding author

E-mail: jiyoung.min@ip-korea.org

Figure S1

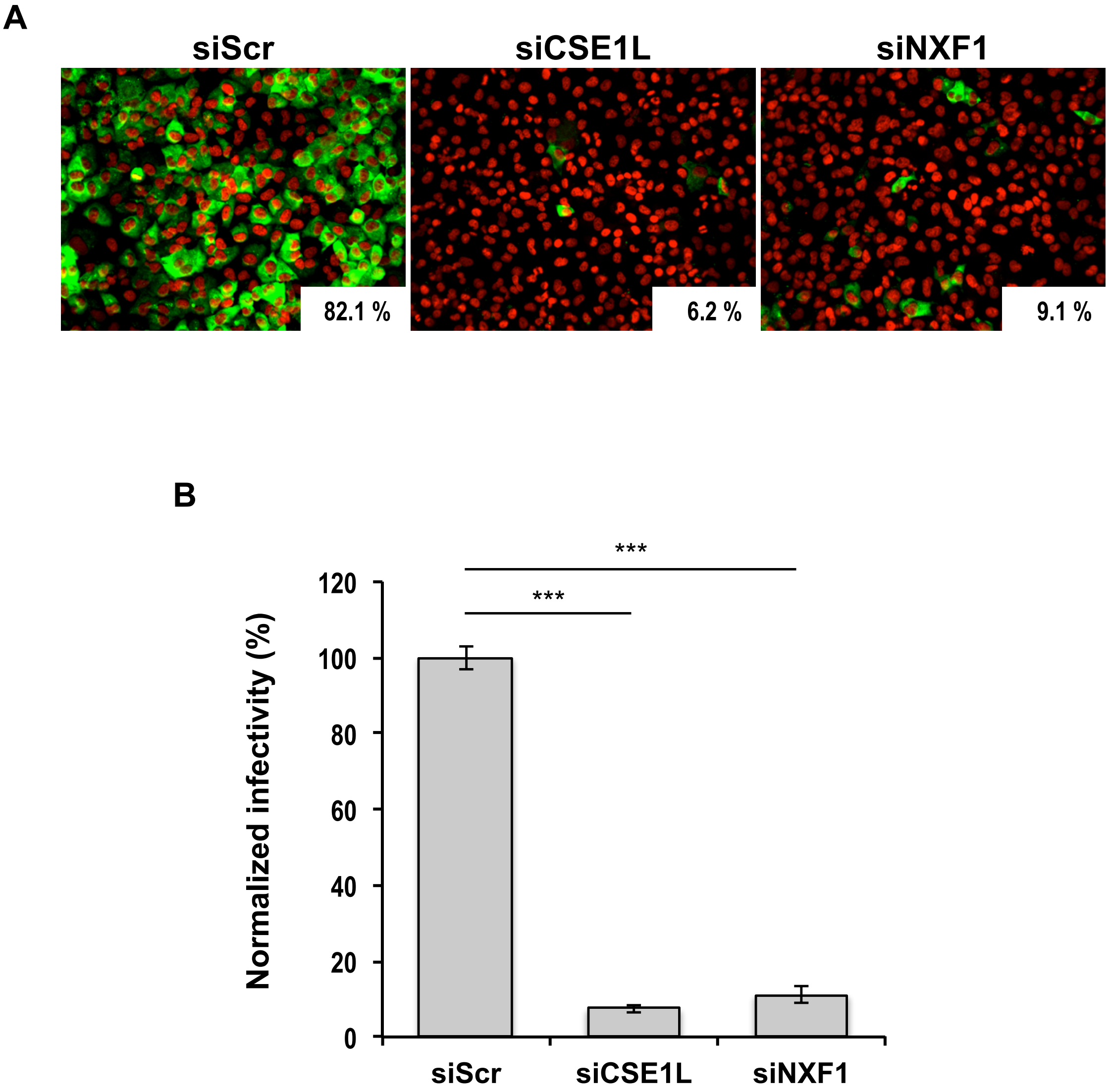
A
siScr
siCSE1L
siNXF1
82.1 %
6.2 %
9.1 %
B
***
***
Normalized infectivity (%)
120
100
80
60
40
20
0
siScr
siCSE1L
siNXF1

Fig S1. Depletion of CSE1L and NXF1 inhibit the viral replication

(A) A549 cells were transfected with 10 nM siCSE1L or siScr and 1 nM siNXF1 48 hours prior to infection. The rPR8-GFP virus was inoculated at an MOI of 5.6. Cells were fixed and stained with Hoechst 33342 10 hours post-infection, and observed with a microplate imaging reader. **(B)** Percentage of infected cells was quantified using in-house IM software. Error bars represent standard deviations. Statistical significance between the indicated groups was tested using the Student's t-test; *** $p<0.001$.
